# The Effect of Trait and State Disgust on Fear of God and Sin

**DOI:** 10.3389/fpsyg.2020.00051

**Published:** 2020-01-29

**Authors:** Patrick A. Stewart, Thomas G. Adams, Carl Senior

**Affiliations:** ^1^Department of Political Science, University of Arkansas, Fayetteville, AR, United States; ^2^Department of Psychology, University of Kentucky, Lexington, KY, United States; ^3^School of Life & Health Sciences, Aston University, Birmingham, United Kingdom

**Keywords:** disgust, scrupulosity, religion, sexuality, Human Behavioral Immune System (HBIS)

## Abstract

There is a growing literature suggesting disgust plays a major role in religiosity. However, the relationships between specific domains of disgust sensitivity and general religious fundamentalism or religious scrupulosity remains unknown and a lack of experimental data prevents the drawing of causal inferences about the potential effects of disgust on religiosity. Two studies are reported that examined the relationship between specific types of disgust sensitivity (i.e., pathogen, sexual, and moral disgust) and specific religious beliefs (i.e., fear of sin and fear of God). In the first study it was found that sexual disgust and pathogen disgust were significantly correlated with fear of sin and fear of God, respectively. In the second study the experimental induction of disgust led to greater fear of sin but not to the fear of God. These findings suggest that pathogen and sexual disgust sensitivities may serve as effective mechanisms for inflated scrupulosity. Taken together the outcomes from both studies converge on a greater understanding of the ‘Human Behavioral Immune System’ model that can account for social behavior with the evolution of adaptive benefit and perhaps more importantly highlights the possible drivers of specific religious behavior.

## Introduction

Even in today’s secular society there is little doubt that the world’s major religions have regulated social behaviors for centuries. There are still a wide range of social behaviors that are regulated in part by religious beliefs such as consumer psychology (Shyan Fam et al., 2004), alcohol consumption (Koopmans et al., 1999) and even organizational decision making (Fernando and Jackson, 2006). Yet despite such a pervasive role in the regulation of our social behavior very little is known about the mechanisms that influence or cause religious scrupulosity.

When considering the Judeo-Christian religion, there are two major factors that are thought to play a major role in motivating and maintaining individual religiosity (Weeden et al., 2008). The first is in-group solidarity, which can provide honest signals of group membership and commitment through complex codes of conduct while also serving as a barrier to out-groups (Fincher and Thornhill, 2008; Thornhill and Fincher, 2014b). The second is reproductive support by providing “low-promiscuity, marriage-centered, heterosexual, high-fertility sexual and reproductive strategies” (Weeden et al., 2008, p. 8). Here, such behavior is sometimes defined as being religious scrupulosity – literally translated to mean the *fearing sin where there is none* – (Abramowitz and Jacoby, 2014). Behaviors that occur as a result of such religious cognitions can be framed as both a fear of moral transgressions, e.g., the fear of committing sins and a fear of a lack of faith and piety, e.g., fear of god (see also Cohen and Rozin, 2001). Within a clinical context religious scrupulosity does have an overlap with obsessive behaviors where patients can manifest with overt behaviors to mitigate the effects of perceived moral transgressions while maximizing their self-perceived piety (Nelson et al., 2006).

In a non-clinical scenario such religious scrupulosity is correlated with conservative or traditional sexual and family attitudes and behaviors with religiosity increasing in the presence of perceived mating competition (Weeden et al., 2008; Li et al., 2010). In other words, religious scrupulosity may operate to prevent risk taking behaviors that might lead, in some circumstances, to pathogen transmission.

Scrupulosity can also be seen to play a major role in religiosity through enhanced self-monitoring of thoughts and behavior. As a result, a religiously scrupulous person is persistently concerned as to whether they have sinned in thought, word, or deed (Abramowitz et al., 2002). Psychometric research has revealed two dimensions of scrupulosity. The first is the fear of sin, in which individuals assess the frequency of their doubts and fears concerning their sins and the degree to which this affects their everyday life. The second dimension is the fear of God, in which the consequences of disobeying God are assessed (Olatunji et al., 2007a, b).

A growing literature has considered the role of disgust on the nature, extent, and expression of religious beliefs (e.g., Ritter et al., 2016). While this literature has focused on disgust sensitivity and general religious fundamentalism (Terrizzi et al., 2010, 2012b; Tybur et al., 2010) or specific forms of religious scrupulosity (Olatunji et al., 2005; Olatunji, 2008), there has been a dearth of research considering the relationship between discrete types of disgust sensitivity or even the emotional state of disgust (as in elevated state disgust) and specific forms of religious beliefs. The studies reported here attempt to address this gap and delineate the role of different types of disgust sensitivity and induced disgust on fear of sin and fear of God.

At first pass it may not be immediately clear how disgust can facilitate religiosity yet as is discussed above religion serves as an adaptive response to environmental threats such as infectious disease. The facilitation of specific reproductive strategies that are operationalized within certain religious codes is an example of a behavioral mechanism of this adaptive response. This class of behaviors can also be described within a theoretical model called the Human Behavioral Immune System (HBIS), which describes behaviors that have a role protecting individuals and groups from pathogens and infections by delineating psychological boundaries that separate the in- and out-group members (Schaller and Murray, 2011; Terrizzi et al., 2012a). There are obvious similarities here with religious organizations that define boundaries that are constructed to maintain a socially conservative value system focusing on “adherence to social norms, in-group cohesion and out-group avoidance” (Terrizzi et al., 2012a, p. 106). Indeed, the HBIS may be seen as operating on individuals through belief and behavior such as promulgating social organizations such as churches, prayer meetings, etc., and thus providing an environmental immune system of sorts; a system of behaviors that prevent contagion within the church by regulating social behavior and providing a social barrier from those outside the church (see Henneberg, 2011; Wiebe, 2013). In other words: “(A)lthough religion apparently is for establishing a social marker of group alliance and allegiance, at the most fundamental level, it may be for the avoidance and management of infectious disease” (Fincher and Thornhill, 2008).

Anti-pathogen behaviors that are promoted by the experience of disgust can foster the separation of members of an in-group population (i.e., the group to which the disgust holder belongs) from members of the outgroup population (i.e., the group which strangers belong). Such a separation would facilitate a general psychological tendency for social conservatism (see e.g., Schaller et al., 2015; Brown et al., 2016). Indeed, there is much evidence supporting the faciliatory role of pathogen stress in the development of social, political and even religious conservatism (e.g., Thornhill et al., 2009; Aarøe et al., 2017). Religious scrupulosity underpins religious fundamentalism yet the mediating role of pathogen stress on scrupulosity has yet to be explored.

Work suggests that disgust is more than a singular defense system that protects the body from pathogens (Rozin et al., 2008). Although multiple models for disgust exist (Rozin and Fallon, 1987; Olatunji et al., 2007c), one recent evolutionary-adaptive framework for disgust may be particularly salient to the understanding of religiosity (Tybur et al., 2009, 2010). Per the evolutionary theory detailed by Tybur et al. (2009), there are three domains for disgust sensitivity; three clusters of situations or contexts in which the basic emotion of disgust operates to facilitate individual, cultural, and evolutionary adaptation to promote survival and reproductive success. This particular model of disgust is comprised of pathogen, sexual, and moral domains of disgust sensitivity (Olatunji et al., 2007c, 2012; Tybur et al., 2009, 2010, 2012)^1^.

The importance of disgust is further reinforced by an overview of the neurological substrates that mediate its experience (see Calder, 2003). In their extensive review of the neurological substrates that underpin the experience of the various forms of disgust Vicario et al. (2017) concluded that distinct forms of disgust may be represented by a number of overlapping and distributed networks that each converge at the anterior insula. This neural substrate has long been seen to play a fundamental role in the perception of facial display of disgust as well as the gustatory experience of bad tastes (Phillips et al., 1997, 1998).

### The Role of Pathogen Disgust and the Fear of God

The importance of cleanliness is a significant feature in nearly all major religious affiliations (Preston and Ritter, 2012). Pathogen disgust likely mediates the rejection of out-groups that potentially pose the threat of contamination (Schaller and Murray, 2011). Thus, religious beliefs concerning purity may be related to negative attitudes toward out-groups including homosexuals (Olatunji, 2008; Inbar et al., 2012; Terrizzi et al., 2012b), foreigners, and immigrants (Navarrete and Fessler, 2006; Hodson and Costello, 2007), and may stem from concern that these groups may potentially transmit pathogens and disease (Tybur et al., 2010). In addition to the direct mediation of attitudes about threatening groups, disgust may operate indirectly through religious beliefs and attitudes (Olatunji, 2008). Core disgust – which is highly akin to pathogen disgust – indirectly influences attitudes toward homosexuals through *fear of sin* and conservative sexual attitudes (Olatunji, 2008). In essence, Christian identification can serve as a marker of in-group conformance with norms of purity (Graham et al., 2009). This in turn can limit the potential for infection by pathogens coming from outsiders. Consistent with this is research suggesting diversity in religious groups increases alongside parasite stress levels (Fincher and Thornhill, 2008; Thornhill and Fincher, 2014a, b). As a result, it is hypothesized that pathogen disgust will predict religious scrupulosity generally and of fear of sin and fear of God specifically.

#### Sexual Disgust and the Fear of Sin

The link to conservative sexual attitudes and disgust is a relatively consistent finding across a wide body of literature (see e.g., Haidt and Hersh, 2001). Here, heightened disgust sensitivity affects behavior to such a degree that the efforts to keep oneself ‘pure’ may actually play a role in the formation of a variety of sexual disorders (Borg et al., 2011). The importance of the relationship between disgust sensitivity and the fear of sin in driving self-regulatory behavior can be seen with the work of Zhong and Liljenquist (2006). Here, it was found that threats to an individual’s moral behavior which would be likely in the case of sexual conservative attitudes resulted in an increase in the perception of sin which also predicted an increase in cleaning behaviors (see also Fetterman, 2016).

Within social organizations such as religions, sexual disgust may be emphasized to avoid mating with individuals that may jeopardize reproductive success (Tybur et al., 2012). Indeed, specific in-group behaviors that are partially regulated by disgust toward out-group sexual behaviors has been shown to increase true paternity (and corresponding reduction in cuckoldry) in offspring (Strassmann et al., 2012). Such reproductive success is an important function of religions, especially as religions tend to champion high fertility and low promiscuity behaviors (Weeden et al., 2008; Li et al., 2010). According to Tybur et al. (2009) religion enhances fertility by setting, monitoring, and enforcing social group norms and values through “avoiding reproductively costly sexual behaviors, narrowing the pool of sexual behaviors and partners to those likely to contribute to the production of healthy viable offspring” (p. 106). At the same time, sexuality specific avoidance due to disgust allows for a range of beneficial social interactions that might have been precluded by pathogen-based disgust (Tybur et al., 2009; Borg and de Jong, 2012) while also limiting potentially reproductively costly within-group behavior. Due to fear of sin apparently being chiefly concerned with self-regulation to maintain in-group stability, it is hypothesized that sexual disgust sensitivity will be positively related with increased fear of sin.

#### Moral Disgust and the Fear of Sin

Moral disgust plays an important role for religious organizations by limiting potentially maladaptive behaviors that disrupt social relationships and their cohesion (Tybur et al., 2012; Chapman and Anderson, 2013; Russell and Giner-Sorolla, 2013). Moral disgust may be seen as most divorced from pathogen avoidance and response, yet utilizes many of the same physiological, psychological, and behavioral responses as pathogen and sexual disgust (Tybur et al., 2012). As a result, it is hypothesized that sensitivity to moral disgust will predict both fear of sin and fear of God.

#### Exploratory Analysis of Anxiety, Anger, Sex, and Religious Identification

Personality traits of anxiety and anger need to be considered in the formation of scrupulosity (Olatunji et al., 2005, 2007a). Trait anger is important for understanding in-group/out-group divisions through aggressive confrontation, not the avoidance propensity seen with disgust. Research considering the three dimensions of disgust has also shown sex or gender differences in response to pathogen, sexual and moral disgust, with females scoring higher in these scales (Tybur et al., 2009, 2010; Olatunji et al., 2012). Furthermore, in a study considering political conservatism, when controlling for the sensitivity to the various forms of disgust as noted above, men had significantly higher levels of religious fundamentalism than women (Tybur et al., 2010). Finally, to examine the effects of personal religious identification, participants were also asked to indicate whether they identified as either Christian or non-Christian.

## The Domains of Disgust and Religion

### Methods

#### Participants

Previous research has regularly reported small effect sizes when studying the associations between disgust sensitivity and conservative attitudes. For example, Inbar et al. (2012) reported partial eta-sq. of 0.02 between DS-R total score and self-reported conservatism. Power analysis using G-Power 3.0 (Faul et al., 2007) suggested that, for the proposed multivariate regression, a minimum sample of 539 subjects would be required to detect a significant effect (*a* = 0.05) with moderate power (0.80) and a small effect size (*R*^2^ = 0.02). A total of 545 adult (18-years or older) undergraduate students enrolled in introductory psychology courses at a large southern American university took part in this study; 523 participants completed all study questionnaires. The average age reported by participants (*n* = 508) was 19.49 (*SD* = 3.29) with a majority female (60.2%), Caucasian/white (87.8%), and belonging to “a Christian religion” (88.1%).

#### Measures

Revised Penn Inventory of Scrupulosity (PIOS-R; Abramowitz et al., 2002; Olatunji et al., 2007a) is a 15-item self-report religious scrupulosity scale that consists of two subscales: the 10-item fear of sin scale that measures fears of having committed a religious sin (e.g., ‘I am afraid of having immoral thoughts’) and the 5-item fear of God scale that measures fears of punishment from God (e.g., ‘I worry that God is upset with me’). Items for this scale are based upon 5-point scales ranging from 0 (‘never’) to 4 (‘constantly’). Internal consistencies for the two scales were strong (Table 1).

**TABLE 1:** Means, standard deviations, alphas and correlations for all measures used in the study.

		*M*	*SD*	α	2	3	4	5	6	7
1.	Fear of Sin – Revised	10.44	7.85	0.93	0.77**	0.17**	0.17**	0.07	0.24**	0.35**
2.	Fear of God – Revised	6.63	4.72	0.90		0.23**	0.13**	0.09*	0.23**	0.23**
3.	Pathogen Disgust	24.74	8.93	0.86			0.60**	0.56**	0.04	0.03
4.	Sexual Disgust	22.94	10.84	0.88				0.56**	−0.17**	0.13
5.	Moral Disgust	25.11	9.19	0.89					–0.09	0.17**
6.	Trait Anger (STAXI)	18.16	4.39	0.79						0.35*
7.	Trait Anxiety (STAI)	46.55	5.63	0.89						

*p < 0.05; **p < 0.01.

Three Domains of Disgust Scale (TDDS; Tybur et al., 2009) is a 21-item self-report scale that measures pathogen (e.g., ‘Standing close to a person who has body odor’), sexual (e.g., Bringing someone you just met back to your room to have sex’), and moral disgust sensitivity (e.g., Forging someone’s signature on a legal document’). Each factor is represented by 7 items that are measured on a zero to 6-point scale, ranging from ‘not at all disgusting’ to ‘extremely disgusting.’ The TDDS has strong psychometric properties (Olatunji et al., 2012) and is gender invariant (Tybur and de Vries, 2013). Internal consistencies of the TDDS factors in the current study were moderate (Table 1).

Trait Anger Scale: The Trait Anger Scale (STAXI-T; Vagg and Spielberger, 1979) is a 10-item self-report scale that measures the degree to which an individual experiences and expresses anger in general (e.g., ‘I feel like hitting someone’). The STAXI-T utilizes a 4-point scale ranging from 1 (‘almost never’) to 4 (’almost always’). The STAXI-T had moderate internal consistency in the present study (Table 1).

Trait Anxiety Scale (STAI-T; Spielberger, 1983) is a 20-item self-report scale that assesses an individual’s general level of anxiety over the past 2 weeks (e.g., ‘I am tense’). The STAI-T uses a 4-point scale ranging from 1 (‘not at all’) to 4 (‘very much so’). Internal consistency of the STAI-T was moderate in the present sample.

#### Procedure

All data were collected through an online questionnaire, an approach as reliable as in-person data collection (Coles et al., 2007 see also Smith and Senior, 2001). All participants provided IRB approved informed consent prior to completing the online protocol and were awarded course credit in exchange for their participation. The orders of questionnaire presentation were randomized.

#### Data Analysis

Bivariate correlations (see Table 1) were carried out prior to multivariate regression. Fear of sin and fear of God were separately regressed onto pathogen, sexual, and moral disgust, trait anger and anxiety, religious status (Christian vs. non-Christian) and gender (see Table 2). All predictors were entered simultaneously. Univariate distributions were normal and checks for multicollinearity revealed no major violations (see Tabachnick et al., 2007).

**TABLE 2:** Religious scrupulosity linear regressions the variance inflation factors (VIF) are also shown.

		Fear of Sin	Fear of God
	VIF	β	β
Pathogen Disgust	1.85	0.05	0.17**
Sexual Disgust	2.44	0.26**	0.06
Moral Disgust	1.76	–0.03	–0.02
Trait Anger (STAXI)	1.21	0.15**	0.16**
Trait Anxiety (STAI)	1.21	0.35**	0.24**
Religion	1.11	0.19**	0.34**
Gender	1.43	−0.21**	−0.12*
*F*-Test (7, 512)		24.93**	24.49**
*R*^2^		0.25	0.25
Adj. *R*^2^		0.24	0.24

*p < 0.05; **p < 0.01.

### Results

#### Fear of Sin

Save for moral disgust, all zero-order correlations with fear of sin were in the expected direction (positive) and statistically significant (Table 1). The fear of sin regression equation was significant and explained 25% of the overall variance (*F*(2,7) = 20.13, *p* < 0.01). All parameters, except pathogen and moral disgust (n/s), reached statistical significance at an alpha level of 5%. With the exception of moral disgust, all parameters functioned in the expected direction. Although moral disgust was associated with lower levels of fear of sin, the zero-order correlation between these two was positive, which suggests the negative beta likely resulted from suppression. When the relative influence of all variables is considered, sexual disgust (β = 0.26, *p* < 0.01), and trait anxiety (β = 0.35, *p* < 0.01) are the most powerful predictors. Higher levels of sexual disgust and trait anxiety are also associated with greater fear of sin. These variables are followed in power by participant gender (β = 0.21, *p* < 0.01), whether the participant identifies as a Christian (β = 0.19, *p* < 0.01), and finally, trait anger (β = 0.15, *p* < 0.01). Males and Christians are more likely to fear sin and higher trait anxiety is related to greater fear of sin.

#### Fear of God

All zero-order correlations with fear of God were in the expected direction (positive) and statistically significant (Table 1). The fear of God regression equation was significant and explained 17% of the variance (*F*(2,7) = 24.49, *p* < 0.01). The resulting model shows that whether or not the respondent identifies as a Christian is the most powerful predictor, with Christians endorsing greater fear of God than non-Christians (β = 0.34, *p* < 0.01). Trait anxiety (β = 0.24, *p* < 0.01) and pathogen disgust (β = 0.17, *p* < 0.01) are significantly and positively related to fear of God as well. Finally, participant gender plays a significant role with males reporting greater fear God than females (β = 0.12, *p* < 0.05).

### Discussion

Results of the first study provide initial evidence that highlights the relationship between specific types of disgust sensitivity and religious fear. In particular, results suggest a positive relationship between two domains of religious scrupulosity and specific domains of disgust sensitivity with sexual disgust predicting a fear of sin and pathogen disgust predicting a fear of God. In the regression model, moral disgust negatively predicts fear of sin. However, as the zero-order correlation between moral disgust and fear of sin was positive and not significant, this effect was likely a statistical artifact. Further work should consider the possible mechanistic role that anger and anxiety play in scrupulosity as trait anger strongly and positively predicted both the fear of sin and the fear of God while trait anxiety positively predicted fear of sin. Taken as a whole, regression models suggest that negative affect likely plays a substantial role in scrupulosity but that specific manifestations of negative affect (e.g., trait anger and disgust sensitivity) are also implicated.

The present study advances the understanding of the relations between individual differences in emotional processing of religion in two ways. First, it provides evidence for specific emotional traits correlating with religious scrupulosity. Second, domain-specific relationships were found between disgust sensitivity and religious scrupulosity. The findings here provide preliminary evidence for the disease avoidance role of specific religious doctrine; sexual disgust predicted fear of sin and pathogen disgust predicted fear of God. It may be that scrupulosity increases due to disgust sensitivity, that disgust sensitivity is elevated because of scrupulous beliefs, or that some as-yet evaluated variables concomitantly influence both. For example, the observed relationships between disgust sensitivity and scrupulosity may be the result of the mostly Christian sample used, given that Christian participants endorsed greater scrupulosity and religious orientation was the most robust predictor of fear of God. This may reflect the fact that many Judeo-Christian-based religions tend to emphasize cleanliness in doctrine and rituals.

This first study highlights the possible relationship between individual differences in specific domains of disgust sensitivity and religious attitudes. If such propensities to disgust are relevant to the manifestation of specific religious attitudes and behaviors, as would be accounted for with the HBIS model, then the acute experience of disgust should also modulate specific religious activity. The second experiment was therefore carried out to assess whether the acute induction of disgust can influence attitudes about religious scrupulosity. Here it was hypothesized that the induction of disgust through pathogen-based pictorial stimuli will have the effect of increasing scrupulosity. More specifically, it was hypothesized that the induction of disgust would increase fear of God based upon the positive associations between this component of religious scrupulosity and pathogen disgust sensitivity. While pathogen disgust sensitivity was not significantly associated with fear of sin when controlling for the effects of other predictors in the regression equation, there remains a significant zero-order correlation between pathogen disgust sensitivity and fear of sin, suggesting that the induction of disgust may also increase fear of sin. This latter prediction is more speculative and is therefore treated as exploratory.

## The Experience of Disgust and Religion

### Participants

Previous work from our group suggests that the experimental induction of disgust can exert large effects on self-reported religious attitudes. For example, an olfactory disgust induction increased self-reported belief in “biblical truth” to a large degree (${\text{η}}_{\text{p}}^{2}$ = 0.10 to 0.18; Adams et al., 2014). The pictorial disgust induction used in the present study was not expected to be as powerful as in-person disgusting odors (see section “Discussion” below). As such, a medium effect size (*d* = 0.50) was used for power analyses, which suggested that, for the proposed mixed ANOVA and moderate power (0.80), a sample of 52 subjects would be required to detect a significant (*a* = 0.05) interaction effect and a sample of 112 subjects would be required to detect a significant between-subjects effect. Two hundred and four individuals entered the experiment’s website, with 175 successfully completing the study. Of this number, 165 accurately identified one of the treatment images as a manipulation check, with 95 participants in the control condition and 70 in the treatment condition. The average age of participants was 25 years old (*SD* = 8.38), with the majority of participants female (78.8%), and identifying as Christian (87.9%).

### Procedures

The entire experimental protocol was delivered online and consisted of the experimental condition in which participants viewed three disgust-inducing images (dog feces, vomit, a cold sore) and a control condition in which three neutral images were presented to participants (a chair, a tree, a mushroom). These stimuli were collected from the public domain and were matched for picture attributes (e.g., complexity etc.) These six images were selected from a total corpus of 14 images, which were first shown to a group of independent raters who were blind to the experimental hypothesis (11 female, with a mean age 19 years within the range 18–21 years). They were asked to rate each image on a 10-point scale for the dimensions of disgust^2^, image complexity and also each the sexual content of each specific images. Of these the disgust images were matched for disgust content (Cold Sore MN = 7.3, *SD* = 1.7, Dog Feces MN = 7.5, *SD* = 2.8, Vomit MN = 7.3, *SD* = 2.4) and image complexity (Cold Sore 1.8, *SD* = 2.3, Dog Feces MN = 1.7, *SD* = 3.0 and Vomit MN = 2.4, *SD* = 1.9). These were distinct from the scores for the control imagery (Chair, Mushroom and Tree) which received an average rating of 1 for image complexity and were considered as having no disgust content at all by the raters. No image was considered to contain any sexual content.

Participants were asked to drag an indicator on a 0–100-point scale with the endpoints ‘Not at all’ and ‘extremely to indicate the degree to which they felt disgusted by each of the experimental stimuli. The procedures were based upon that used by Smith et al. (2011) with participants viewing each image for 10 s apiece, with 5 s between each image. To verify participants were complying with the task they were asked to submit a description of one of the images they saw. Participants were also asked to complete the PIOS-R and TDDS items as described above. As with study 1 all possible orders of presentation were randomized throughout.

### Data Analysis

Independent samples *t*-tests were performed to examine the effect of the image-based disgust induction on subjective disgust ratings. Mixed-factor ANOVA was then used to test the effects of the treatment (control vs. disgust induction) on scrupulosity (main effect of treatment) and the effects of the treatment on specific domains of scrupulosity (treatment by PIOS factor interaction). Independent samples *t*-tests were performed to probe significant interaction effects.

### Results

After adjusting for the violation of homogeneity, there were significant differences in subjective disgust ratings, *t*(163) = 11.68, *p* < 0.001, between the treatment (*M* = 68.03, *SD* = 38.51) and control (*M* = 8.50, *SD* = 21.35) conditions. Here the experimental stimuli that were used in this study powerfully induced the experience of disgust with a Cohen’s *d* of 1.91.

**FIGURE 1:**
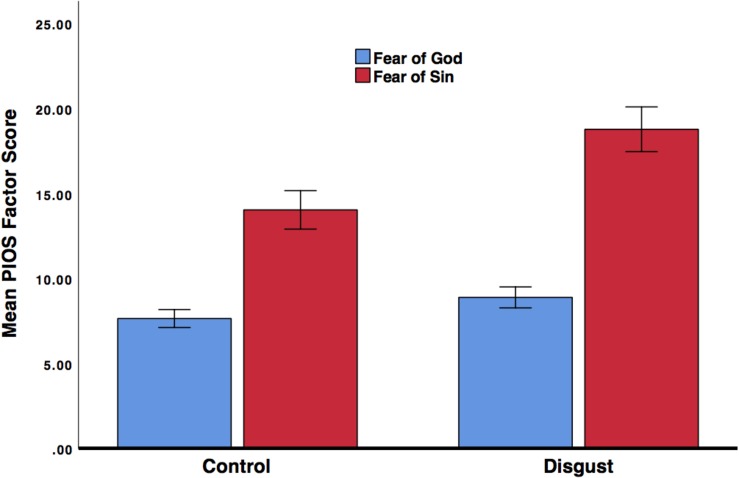
The treatment effects on fear of God and fear of sin scrupulosity domains for the experience of disgust.

There was a significant main effect of the treatment on scrupulosity, *F*(1,163) = 7.20, *p* < 0.01, partial-*η*^2^ = 0.04, suggesting that the disgust induction increased scrupulosity (control *M* = 10.84, *SE* = 0.73 and disgust *M* = 13.83, *SE* = 0.85). There was also a significant interaction between the treatment and PIOS dimension factor, *F*(1,163) = 5.17, *p* < 0.05, partial-*η*^2^ = 0.03 (see Figure 1). *Post hoc* contrasts revealed that fear of God, which due to the findings of Study 1 was hypothesized to be increased by pathogen disgust induction, was not significantly different between participants in the disgust and control groups; though results were trending in the hypothesized direction, *t* = 1.53, *p* = 0.13, *d* = 0.24 (disgust group *M* = 8.89, *SD* = 5.55; control *M* = 7.64, *SD* = 4.85). On the other hand, participants in the pathogen disgust induction group did report significantly greater *fear of sin* than participants in the control group, *t* = 2.73, *p* < 0.01, *d* = 0.42 (treatment *M* = 18.77, *SD* = 13.17; control *M* = 14.03, *SD* = 9.11). There were no significant between group differences in sex, age, religion, moral disgust, sexual disgust, or pathogen disgust (all *p* > 0.10).

### Discussion

The major findings are that the acute induction of disgust had a significant effect on scrupulosity and this effect was significantly pronounced for the fear of sin, but not the fear of God. These latter findings are somewhat unexpected given the findings from Study 1; regression analyses revealed a significant relationship between individual differences in pathogen disgust sensitivity and fear of God but not fear of sin. However, significant zero-order correlations were observed between individual differences in pathogen disgust and fear of sin and fear of God. One of the three images used to induce disgust in the second study showed an open sore under the lips of the model, which could be linked to sexual activity, and may have activated sexual disgust; however, this is unlikely given that all independent raters rated all three disgust induction pictures as having zero sexual content. The stronger effect of the disgust induction may be explained by the nature of the threats posed by the images in the disgust treatment condition. Specifically, feces, vomit, and open sores are addressed on a regular basis within social groups. Thus the connection with fear of sin may be expected due to that dimension’s focus on the self-monitoring of everyday behavior.

## General Discussion

Researchers are increasingly appreciating the role played by disgust in religiosity, especially given the central role religion plays in informing social and political attitudes (Olatunji et al., 2007c; Olatunji, 2008; Thornhill and Fincher, 2014b). This study adds to and elaborates on the role played by disgust sensitivity and its induction on religious scrupulosity at the individual level, finding that not only do different types of disgust sensitivities play a role in specific types of religious scrupulosity, with fear of sin most strongly related to with sexual disgust sensitivity and fear of God with pathogen disgust sensitivity, but also that experimentally induced disgust influences religious scrupulosity, albeit at different levels for fear of sin and fear of God.

The implications of our findings are that higher levels of pathogen and sexual disgust sensitivity may play an important role in religious attitudes related to scrupulosity. With regard to the relation between sexual disgust sensitivity and fear of sin, both studies provide preliminary evidence for the disease avoidance role of proscribed behavior within religions. Results from these studies may be due, at least in part, to the mostly Christian sample used in this study. As many of the prohibited or sinful practices within Judeo-Christianity are sexual in nature, it may also be that sexual practices in general come to be associated with disgust through evaluative or other verbal forms of conditioning (Olatunji et al., 2007b). This alternative explanation may be even more viable, given that some items on the sexual subscale of the TDDS pose relatively little disease risk.

Although the current study provides strong evidence for the relation between trait and state disgust and religious scrupulosity, findings should be interpreted with caution due to several limitations. The relatively young and demographically homogenous sample limits the generalizability of both studies. Furthermore, the overwhelming majority of participants in both studies identified as Christian. As such, findings may not generalize to individuals of other religious backgrounds. Similarly, the measure for religious scrupulosity is largely based on a monotheistic perspective. It remains to be seen whether a more diverse religious perspectives both in sampling and measurement would result in a different outcome. While the findings from both correlational and experimental designs underscore the role of disgust both as a trait and an induced state, effect sizes were small to medium. Moreover, replication is necessary to more strongly support and accurately delineate the relationships between disgust and religious scrupulosity.

Geography may have also influenced the present results. Specifically, participants were drawn from a population tending to be raised and currently living in the states comprising the American South. This region is noted for being highly conservative, which according to Thornhill and Fincher (Thornhill and Fincher, 2014a) has beliefs that may be seen as partly driven by an “ideological defense against infectious diseases” (p. 6). In other words, many participants in both studies, in addition to being exposed to religious and cultural teachings that focus on in-group favoritism and out-group avoidance, as well as concomitant strategies of favoring tradition while avoiding new ideas and practices, may also have an accentuated response to pathogenic threat.

Further studies should consider the possible implications of the relatively large sample size for the first study, which was determined on the basis of previous work that had employed relatively large cohorts to examine disgust sensitivity and its predictive power for a variety of complex social processes that are similar to religious scrupulosity (Inbar and Gilovich, 2011). Such an approach is valid yet needs to be replicated with large and demographically representative samples to exclude the possibility of a false positive result (Forstmeier et al., 2017).

One further note for future work to consider is based on the theoretical and empirical premise of the work of Thornhill and colleagues (e.g., Fincher and Thornhill, 2008), which employed group wide analyses with countries or regions as units of analysis to elucidate the role of pathogen stress in the facilitation of in-group behaviors. It is worth noting that some scholars have argued that such a “Nation-unit analyses always suffer(s) from Galton’s problem, in which units of analysis fail to ensure statistical independence” (Horita and Takezawa, 2018, p. 2). Such an issue can be addressed by grouping the units of analysis along shared demography (Murray, 2014). Indeed, future studies that integrate geographic factors into their exploration of the relationship between pathogen stress and religious scrupulosity should be mindful of these types of artifacts.

Taken together the findings from both studies inform our understanding of the Human Behavioral Immune System model and highlight the means by which this theoretical model can account for idiosyncratic religious attitudes. Results from these studies highlight the nuanced ways that individual differences in specific domains of disgust sensitivity and the acute induction of a disgusted state can influence distinct manifestations of scrupulosity.

## Data Availability Statement

The datasets generated for this study are available on request to any of the corresponding authors.

## Ethics Statement

The study was reviewed and approved by the University of Arkansas IRB (Ref 12-02-494). All participants provided written informed consent.

## Author Contributions

All authors listed have made a substantial, direct and intellectual contribution to the work, and approved it for publication.

## Conflict of Interest

The authors declare that the research was conducted in the absence of any commercial or financial relationships that could be construed as a potential conflict of interest.

## Footnotes

1 Anecdotal evidence also suggests a possible link between the fear of sin as a regulatory social behavior within the finance industry (Hirshleifer, 2008).

2 Raters were asked to look at each picture and decide how disgusting, complex or sexual each scene was and disgust images retained were those that scored within 1 SD of each other. We would like to thank the reviewer for prompting us to include this here.
